# The value of radiomics features of white matter hyperintensities in diagnosing cognitive frailty: a study based on T2-FLAIR imaging

**DOI:** 10.1186/s12880-025-01732-y

**Published:** 2025-05-22

**Authors:** Qinmei Liao, Xihao Hu, Zhiqiong Jiang, Xiaoyun Huang, Jiacheng Guo, Yuanzhong Zhu, Wenjing He

**Affiliations:** 1 School of Medical Imaging, North Sichuan Medical College, Nanchong, 637000 China; 2 Department of Gerontology, Affiliated Hospital of North Sichuan Medical College, Nanchong, 637002 China

**Keywords:** White matter hyperintensities, Cognitive frailty, Radiomics, T2-FLAIR

## Abstract

**Background:**

White matter hyperintensities (WMHs) are closely associated with cognitive frailty (CF). This study aims to explore the potential diagnostic value of WMHs for CF based on radiomics approaches, thereby providing a novel methodology for the early diagnosis and timely intervention of CF.

**Methods:**

The present study conducted a retrospective analysis on 147 patients (77 with CF, 70 in the control group). Following an 8:2 ratio, the patients were randomly divided into training and testing sets. Repeated 5-fold cross-validation was adopted for model training and evaluation. Optimal radiomic features were extracted and selected from T2-FLAIR images, and multiple logistic regression analysis was utilized to identify independent risk factors. Three machine learning algorithms—K-Nearest Neighbors (KNN), Logistic Regression (LR), and Support Vector Machine (SVM)—were used to construct radiomic models, clinical models, and combined models. The performance of each model in diagnosing CF was evaluated using metrics including the area under the curve (AUC), area under the net benefit curve (AUNBC), and Brier score.

**Results:**

In the test set, the AUC values of KNN, LR, and SVM in the radiomics models were 0.860, 0.916, and 0.885, respectively; the AUC values of the clinical models were 0.868, 0.850, and 0.787, respectively; and the AUC values of the combined models were 0.906, 0.954, and 0.930, respectively. The decision curve analysis (DCA) demonstrated that the combined model was superior to the single models in terms of clinical decision-making efficacy.

**Conclusion:**

The radiomic model, clinical model, and combined model can effectively diagnose CF patients, with the combined model demonstrating the best diagnostic efficacy.

**Clinical trial number:**

Not applicable.

**Supplementary Information:**

The online version contains supplementary material available at 10.1186/s12880-025-01732-y.

## Introduction

With the intensification of global population aging, cognitive impairment and frailty have become two important components among the four major “geriatric giants” in modern times [1]. Cognitive frailty (CF) refers to the simultaneous presence of physical frailty and cognitive impairment in older adults, excluding dementia [2]. In investigations across 17 countries or regions, the prevalence of CF in the elderly population exhibits significant geographical variations, ranging from 1 to 50% [3, 4]. CF not only significantly increases the risk of progression to dementia but is also closely associated with falls, disability, and mortality. However, cognitive frailty presents potential reversibility, making it a crucial target for secondary interventions in asymptomatic stages and early dementia [5].

Currently, the diagnosis of CF primarily relies on clinical scales such as the Mini-Mental State Examination (MMSE) and the Fried Frailty Phenotype (FP). These traditional methods have significant limitations: they are time-consuming and constrained by the cognitive and physiological state of the individual at the time of testing, making early warning difficult to achieve. Research has confirmed that the volume of white matter hyperintensities (WMHs) is significantly associated with cognitive impairment [6], and pathological changes may exist years before a detectable decline in cognitive function on these scales. In studies investigating the pathological mechanisms of CF, WMHs have garnered considerable attention as important imaging biomarkers. WMHs are typically observable in T2-weighted imaging (T2WI) or T2 fluid-attenuated inversion-recovery (T2-FLAIR) sequences, presenting as punctate or patchy hyperintensities in the bilateral periventricular or subcortical white matter regions. WMHs are typically associated with cerebral small vessel diseases, degenerative diseases, leukodystrophies, and demyelinating inflammatory diseases [7]. The occurrence of WMHs is closely related to age, with 87% of individuals aged 60–70 years having WMHs, and 100% of participants aged 80–90 years exhibiting WMHs [8]. WMHs are considered important factors that increase the risk of cognitive impairment and frailty, as they disrupt long-range white matter fibers, affecting the efficiency of information transmission between brain regions and consequently leading to a decline in cognitive abilities [9, 10]. More importantly, recent studies have confirmed that WMHs are imaging markers of neurodegeneration and can predict cognitive decline in the general population [8].

In the study of WMHs, most research employs the Fazekas scale for visual grading or utilizes ITK-SNAP software and related segmentation algorithms for volume measurement [11–14]. Although these methods have some value in analyzing the correlation between WMHs and cognitive impairment or frailty, they remain somewhat superficial and fail to fully explore the potential information contained in WMHs.

In recent years, the rapid development of radiomics technology has provided new possibilities for the in-depth study of WMHs. By extracting high-throughput quantitative features from medical images and combining them with machine learning algorithms for automated analysis, radiomics has demonstrated significant advantages in the diagnosis and prediction of neurological diseases such as depression, epilepsy, and Alzheimer’s disease [15–17]. Particularly in the field of cognitive impairment, radiomics technology has shown tremendous application potential [18, 19]. However, there is currently limited research on the radiomic features of WMHs in CF, and no studies have yet explored the value of combining radiomic features with clinically independent risk factors in the diagnosis of CF.

Therefore, this study aims to construct a diagnostic model by extracting radiomic features from the WMHs region and integrating clinical independent risk factors, addressing the limitations of traditional scales in early warning and quantitative assessment. This study was designed and reported in accordance with the CLEAR and METRICS guidelines (Supplementary Material 1 and 2).

## Materials and methods

### Research subjects

This retrospective study was approved by the Institutional Review Board, and the requirement for obtaining informed consent was waived. MRI images of 243 patients who underwent brain MRI scans and cognitive frailty assessments at our hospital from August 2016 to June 2023 were retrospectively collected. Cognitive frailty was evaluated using the MMSE and the FP, covering aspects such as memory, language, and mobility. Grouping criteria: The case group consisted of patients with MMSE scores ranging from 18 to 26 and FP ≥ 1 [12], while the rest were assigned to the control group. Inclusion criteria: (1) Age ≥ 60 years old; (2) The presence of white matter hyperintensities on magnetic resonance images; (3) Complete assessment data. Exclusion criteria: (1) Age < 60 years old; (2) Incomplete clinical or imaging data of the study subjects; (3) The presence of artifacts in magnetic resonance images; (4) Patients diagnosed with dementia.

A total of 147 patients were ultimately included in the study (The minimum sample size is provided in Supplementary file 3), consisting of 77 patients with cognitive frailty and 70 in the control group. To enhance the model robustness, the dataset was randomly partitioned into training and test sets at an 8:2 ratio using a random sampling approach. Within the training set, repeated 5-fold cross-validation was employed for data splitting. Specifically, the outer loop was constructed through five rounds of data shuffling, followed by 5-fold cross-validation as the inner loop during each iteration, thereby comprehensively evaluating the stability of model performance. The detailed workflow is illustrated in Supplementary Figure S1. Furthermore, basic clinical data, including age, gender, hypertension, and diabetes, were collected from the hospital information system.

### MRI image acquisition

The MRI scans were carried out using a GE Signa HDx 3.0T MRI (GE Medical Systems, USA) equipped with a standard 8-channel head coil for data acquisition. Prior to scanning, to protect the patients’ hearing, noise-reducing earplugs were assisted to be worn by the patients. All patients were placed in the supine position and their heads were fixed with sponge pads to reduce the impact of motion artifacts. During the scanning process, axial T1-weighted imaging (T1WI), T2WI, and T2-FLAIR were performed in sequence. (1) For the acquisition of three-dimensional high-resolution T1WI, an inversion recovery-steady-state gradient echo sequence was employed. The scanning parameters were as follows: TR 8.3ms, TE 3.3ms, flip angle 12°, field of view 24 cm×24 cm, number of excitations 1, reconstruction matrix 256 × 256, slice thickness 1.0 mm, and slice gap 0 mm. (2) For the T2WI sequence: TR 3780ms, TE 76.6ms, field of view 24 cm×24 cm, slice thickness 5.0 mm, slice gap 1.0 mm, flip angle 110°, number of excitations 1, echo train length 30, and bandwidth 83.33 Hz.(3) For the T2-FLAIR sequence: TR 5000ms, TE 388ms, field of view 24 cm×24 cm, reconstruction matrix 256 × 256, and slice thickness 1.0 mm. All scans were positioned at the mid-sagittal plane, with the central line parallel to the anterior-posterior commissure line.

### Region of interest segmentation

T2-FLAIR images of the patients were imported into 3D Slicer 5.0.3 software (https://www.slicer.org/). Image analysis was performed by a radiologist (Physician A) with over six years of experience. Prior to segmentation, physicians were blinded to the patients’ CF status to minimize potential bias. The Level Tracing tool within the software was first utilized by positioning the mouse pointer within white matter hyperintensity (WMH) regions, enabling automated identification and delineation of contiguous areas with signal intensity similar to the selected reference point. Subsequently, the physician manually adjusted and optimized the regions of interest (ROIs) based on the automatic identification to ensure that the ROI encompassed the entire lesion area. Given the uncertainty of the boundaries of the white matter hyperintensities, all ROI delineations were repeatedly confirmed and corrected in the axial, sagittal, and coronal planes. The white matter segmentation map can be found in Supplementary Figure S2.

### Consistency analysis and feature extraction

Thirty patient images were randomly selected. Radiologist A, along with another radiologist (Radiologist B) boasting eight years of professional experience, delineated the ROIs following the aforementioned approach. After a one-month interval, Radiologist A repeated the ROI delineation for the same set of samples. The Intraclass Correlation Coefficient (ICC) was utilized to evaluate the intra-observer and inter-observer consistency. A feature consistency was considered to be satisfactory when the ICC value was greater than 0.75.

Prior to feature extraction, the images were resampled to 1 mm × 1 mm × 1 mm using the bilinear interpolation method. The original images were processed using various filters, including the Laplacian of Gaussian filter, recursive Gaussian filter, and wavelet transform filter. This study utilized the PyRadiomics toolkit (v3.0.1) on the Python platform to automatically extract radiomics features from regions of interest (ROIs). Detailed parameter configurations for feature extraction are provided in the Supplementary file 4. A total of 1,520 features were ultimately extracted, encompassing shape features, texture features, and gray-level features.

### Feature selection

Before the selection of imaging features, to avoid bias in subsequent analyses due to outliers in radiomic features, this study employed Winsorization based on the interquartile range (IQR) for correction. Subsequently, all features were standardized using Z-score normalization to eliminate the potential impact of dimensional differences among different features on the model results. Independent - samples t - test was carried out on the features with ICC>0.75, and the features with statistical significance (*p* < 0.05) were screened out. Least absolute shrinkage and selection operator (LASSO) regression combined with 10-fold cross-validation was applied to the training set data to identify features significantly associated with the target variable and calculate their corresponding coefficients. The imaging features with feature coefficients greater than 0 were retained and sorted in descending order of the absolute value of the feature coefficients. Finally, the features were incorporated into Logistic Regression (LR) one by one for feature screening until the area under the curve (AUC) of the model reached the maximum value. The corresponding feature was regarded as the optimal feature. The basic clinical characteristics were analyzed using univariate logistic regression to identify statistically significant features (*P* < 0.05), which were then included in multivariate logistic regression analysis to determine the independent risk factors affecting CF.

### Model construction

Three machine learning algorithms—k-nearest neighbor (KNN), LR, and support vector machine (SVM)—were utilized to construct three types of models: a radiomic model based on the optimal radiomic features; a clinical model based on independent risk factors; and a combined model (radiomic-clinical model) that integrates independent risk factors and radiomic features. The machine learning model has been optimized through parameter tuning with the following settings: (1) KNN: n_neighbors = 5, algorithm = ‘auto’. (2) LR: class_weight = ‘balanced’, C = 1.0. (3) SVM: class_weight = ‘balanced’, kernel = ‘linear’, C = 0.1. All other parameters retained their default settings.

### Model evaluation

**Fig. 1:**
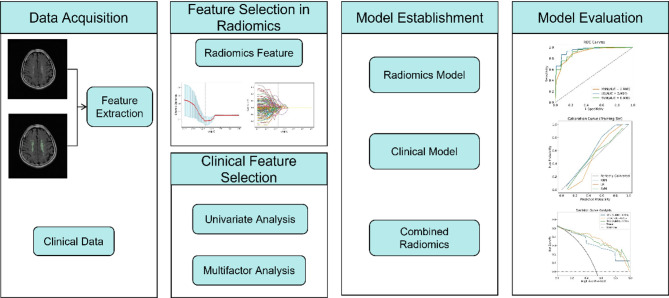
Flowchart of this study

The diagnostic performance of the models was assessed using the area under the curve (AUC), accuracy, specificity, sensitivity, and F1-score. To compare the differences in AUC values between models, DeLong’s test was performed. Additionally, clinical decision curve analysis (DCA) and the area under the net benefit curve (AUNBC) were used to assess the clinical net benefit at different thresholds. The calibration performance was assessed using calibration curves and the Brier score. The workflow diagram is shown in Fig. 1.

### Statistical analysis

Statistical analysis was performed using SPSS software version 26.0 and Python software version 3.10.9. Categorical data were presented as frequency and percentage and analyzed using the χ² test or Fisher’s exact test. Quantitative data were expressed as mean ± standard deviation and tested for normality using the Kolmogorov-Smirnov test. For data that met the assumptions of normal distribution and homogeneity of variance, independent samples t-tests were used for analysis; for data that did not meet these assumptions, the Wilcoxon rank-sum test was applied.

## Results

### Clinical information

There were statistically significant differences between the cognitive frailty group and the control group in terms of age, education level, community activities, household labor, clock drawing test (CDT), and diabetes (*P* < 0.05). However, no statistically significant differences were observed regarding sex, marital status, residence, residential status, nutritional status, or the presence of coronary artery disease, osteoporosis, and hypertension, as shown in Table 1. After conducting univariate logistic regression analysis, the clinically significant features were included in multivariate logistic regression analysis. The results indicated that age, education level, community activities, household labor, clock drawing test, and diabetes were independent risk factors, as detailed in Table 2.

**Table 1:** Comparative analysis of general data between cognitive frailty and control group

Clinical data	Training set (*n* = 117)		*P*	Test set(*n* = 30)		*P*
	CF(*n* = 62)	non-CF (*n* = 55)		CF(*n* = 15)	non-CF(*n* = 15)	
Age	80.08 ± 7.02	72.95 ± 7.17	0.000*	82.53 ± 8.62	72.40 ± 8.98	0.004*
Sex			0.708			0.710
Male	25(40.32)	25(45.45)		5(33.33)	7(46.67)	
Female	37(59.68)	30(54.55)		10(66.67)	8(53.33)	
Marital status			0.126			0.390
Married	44(70.97)	46(83.64)		10(66.67)	13(86.67)	
Divorced and widowed	18(29.03)	9(16.36)		5(33.33)	2(13.33)	
Residence			0.086			0.483
Rural areas and suburbs	6(9.68)	2(3.64)		1(6.67)	0(0.00)	
Counties, towns and cities	11(17.74)	4(7.27)		1(6.67)	0(0.00)	
Large and medium-sized cities	45(72.58)	49(89.10)		13(86.67)	15(1.00)	
Educational level			0.000*			0.008*
Illiterate	5(8.10)	0(0.00)		0(0.00)	0(0.00)	
Primary school	17(27.41)	0(0.00)		7(46.67)	0(0.00)	
Middle school	33(53.22)	34(61.82)		5(33.33)	7(46.67)	
University and above	7(11.29)	21(38.18)		3(20.00)	8(53.33)	
Residential status			0.118			0.662
Live alone	3(4.84)	6(10.91)		3(0.20)	3(0.20)	
Live with children	18(29.03)	8(14.55)		3(0.20)	1(6.67)	
Live with spouse	41(66.13)	41(74.55)		9(0.60)	11(73.33)	
Community activities			0.004*			0.002*
Never	19(30.65)	5(9.09)		4(26.67)	1(6.67)	
Seldom or occasionally	30(48.39)	27(49.09)		10(66.70)	4(26.7)	
Often	13(20.97)	23(41.82)		1(6.70)	10(66.7)	
Nutritional status			0.066			0.169
Normal	41(66.12)	45(81.82)		10(66.67)	14(93.33)	
At risk of malnutrition	17(27.42)	10(18.18)		5(33.33)	1(6.67)	
Poor	4(6.45)	0(0.00)		0(0.00)	0(0.00)	
Household chores			0.000*			0.052
Never	27(43.55)	3(5.45)		4(26.7)	0(0.00)	
Occasionally	14(22.58)	13(23.64)		5(33.3)	3(20.0)	
Often	21(33.87)	39(70.91)		6(40.0)	12(80.0)	
CDT			0.000*			0.000*
0	2(3.23)	0(0.00)		1(6.67)	0(0.00)	
1	3(4.84)	0(0.00)		5(33.33)	0(0.00)	
2	14(22.58)	1(1.82)		5(33.33)	1(6.67)	
3	20(32.26)	14(25.45)		4(26.67)	14(93.33)	
4	23(37.10)	40(72.73)				
CAD			0.440			0.715
Absent	38(61.29)	38(69.10)		6(0.40)	8(53.33)	
Present	24(38.71)	17(30.91)		9(0.60)	7(46.67)	
Osteoporosis			0.337			0.682
Absent	37(59.68)	38(69.10)		10(66.67)	12(0.80)	
Present	25(40.32)	17(30.91)		5(33.33)	3(0.20)	
Diabetes			0.034*			0.427
Absent	34(54.84)	41(74.55)		12(0.80)	9(0.60	
Present	28(45.16)	14(25.45)		3(0.20)	6(0.40)	
Hypertension			1.000			1.000
Absent	19(30.65)	16(29.10)		8(53.33)	6(0.40)	
Present	43(69.35)	39(70.90)		7(46.67)	9(0.60)	

*CDT* Clock Drawing Test; *CAD* Coronary Artery Disease; *p<0.05

**Table 2:** Statistical analysis of clinical independent risk factors

Characteristic	Univariate logistic regression analysis		Multivariate logistic regression analysis	
	OR(95%CI)	*P*	OR(95%CI)	*P*
Age	1.153(1.083–1.228)	0.000*	1.260(1.118–1.420)	0.000*
Sex	1.233(0.592–2.570)	0.576		
Marital status	2.091(0.850–5.146)	0.108		
Residence	0.467(0.223–0.977)	0.043*	0.637(0.161–2.512)	0.519
Educational level	0.165(0.074–0.369)	0.000*	0.041(0.007–0.223)	0.000*
Residential status	0.941(0.525–1.688)	0.869		
Community activities	0.405(0.230–0.714)	0.002*	0.216(0.069–0.679)	0.009*
Nutritional status	2.390(1.098–5.205)	0.028*	2.389(0.646–8.827)	0.192
Household chores	0.290(0.169–0.497)	0.000*	0.406(0.175–0.942)	0.036*
CDT	0.278(0.151–0.514)	0.000*	0.318(0.105–0.957)	0.041*
CAD	0.378(0.656–3.040)	0.378		
Osteoporosis	1.510(0.703–3.244)	0.290		
Diabetes	2.412(1.099–5.294)	0.028*	7.388(1.627–33.559)	0.010*
Hypertension	0.928(0.420–2.054)	0.855		

*CDT* Clock Drawing Test; *CAD* Coronary Artery Disease; *OR* Odds ratio; *95%CI* 95% confident interval; *P<0.05

### Feature extraction and selection

**Fig. 2:**
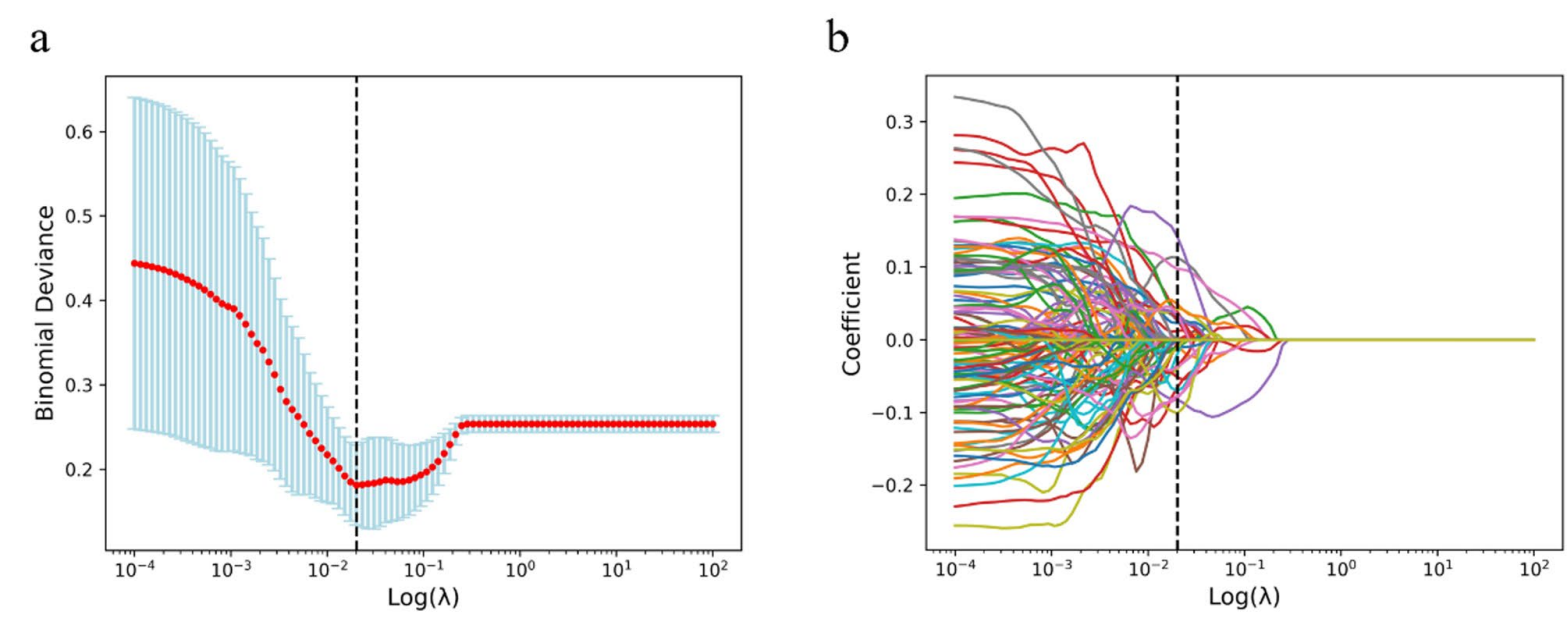
LASSO algorithm for selecting radiomic features (**a**), This figure describes the model fitting performance under different log(λ) values (**b**), This figure illustrates the variation of feature coefficients with log(λ) values

**Fig. 3:**
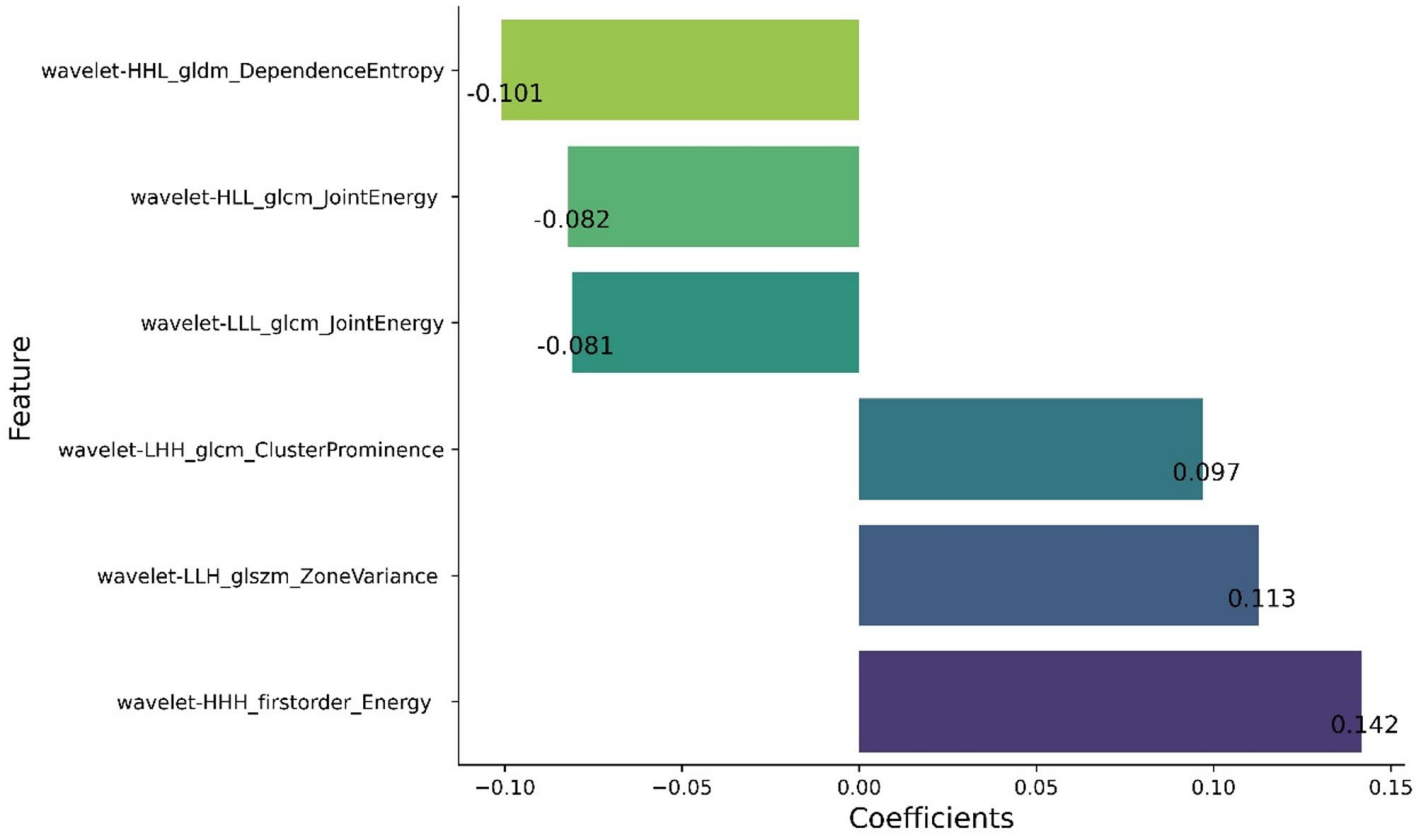
Optimal radiomic features

A total of 1520 features were extracted from the T2-FLAIR images. Through consistency testing, the inter-group ICC value was 0.678 ± 0.240, while the intra-group ICC value was 0.681 ± 0.258. Features with an ICC value greater than 0.75 were retained, resulting in the selection of 528 characteristics with good consistency. Further analysis using independent samples T-test identified 489 characteristics with statistical significance (*P* < 0.05). Subsequently, using the LASSO algorithm (Fig. 2) and the LR algorithm, six optimal imaging features were screened out, as shown in Fig. 3.

### Model comparison

**Fig. 4:**
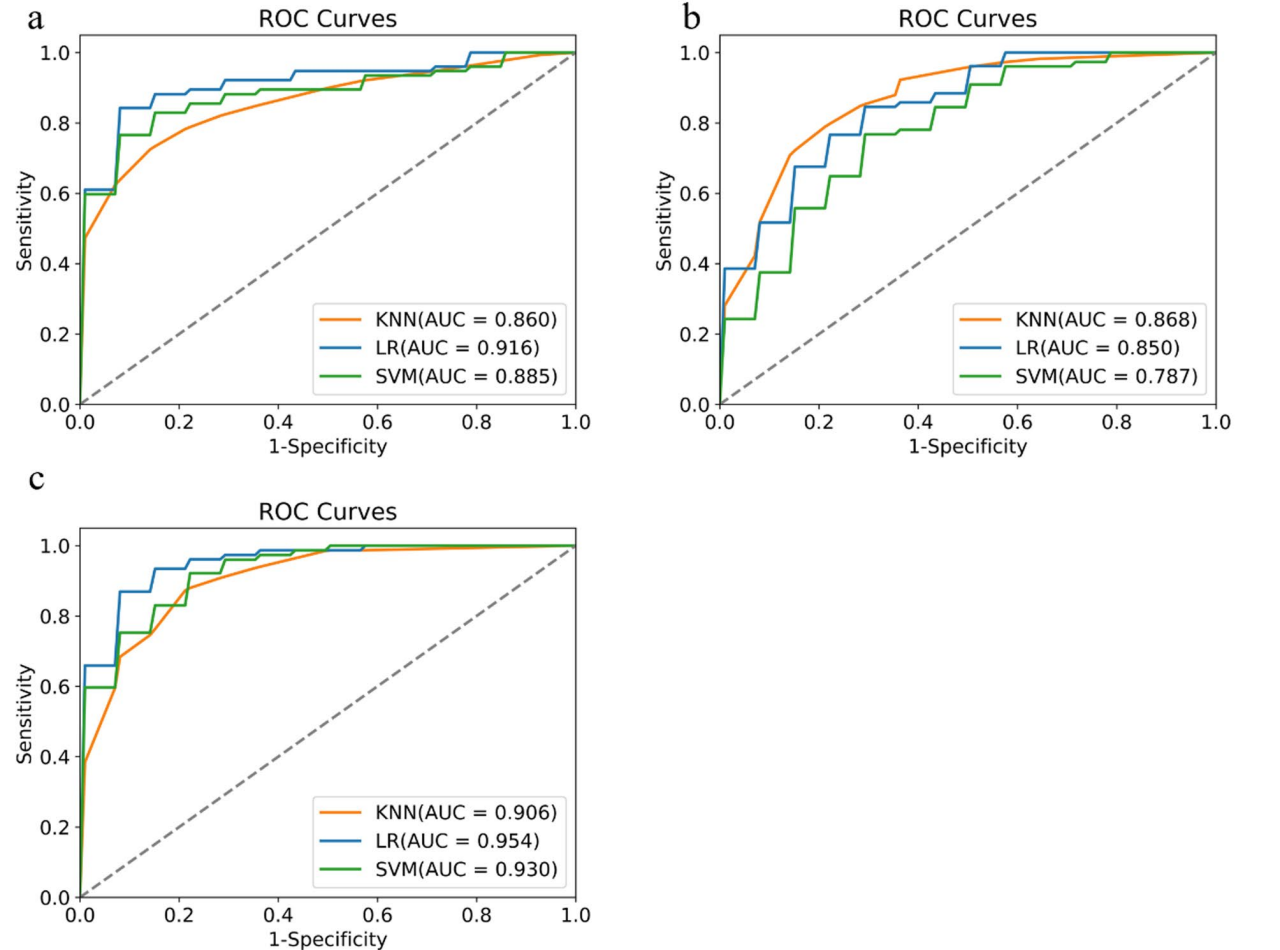
Evaluation of model performance in the test set (**a**) ROC of the radiomic model (**b**) ROC of the clinical model (**c**) ROC of the combined model

**Fig. 5:**
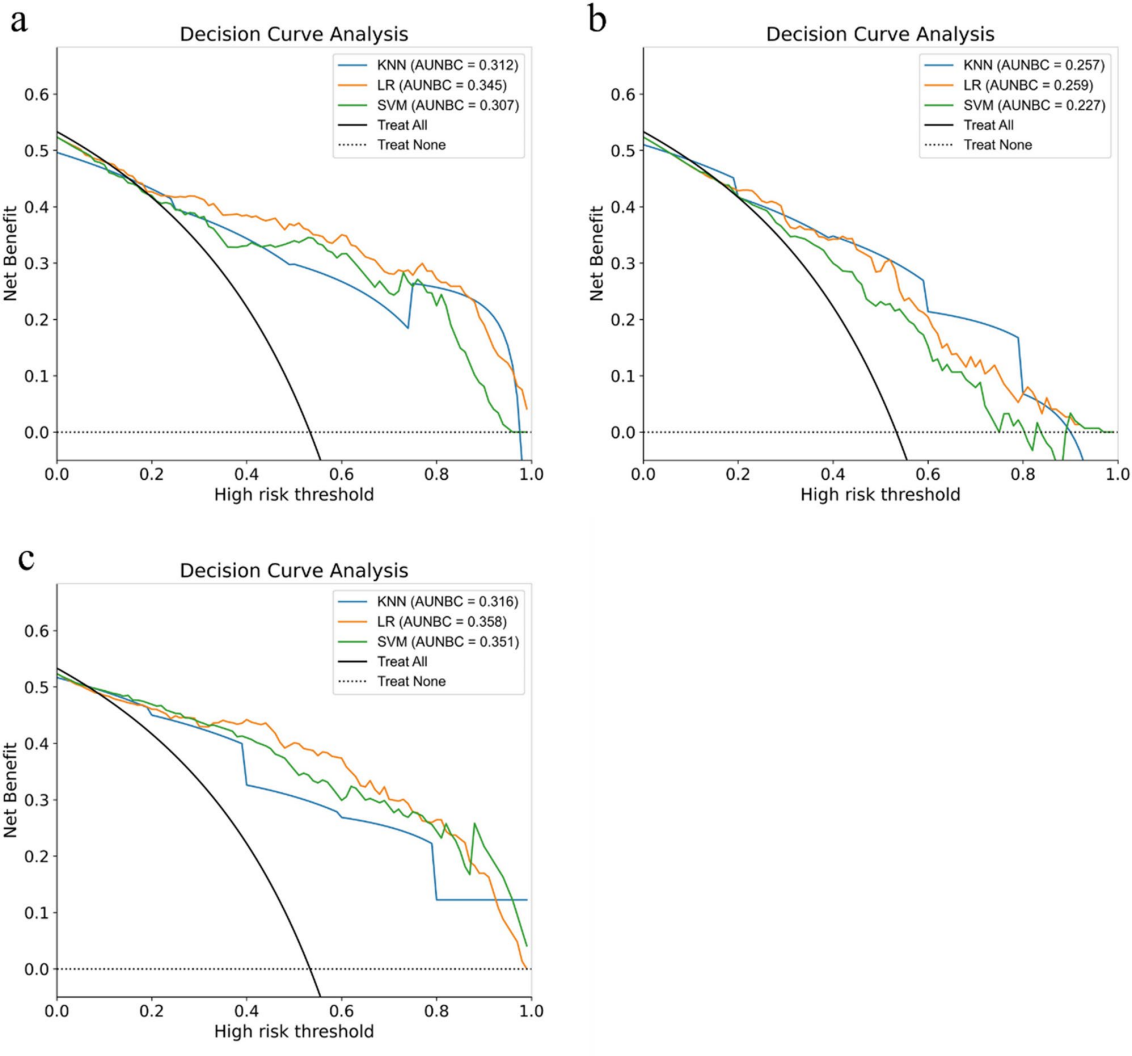
Clinical decision curve analysis (DCA) for the test set The solid line represents the mean net benefit, and the shaded area indicates the standard deviation. (**a**) DCA of the radiomic model (**b**) DCA of the clinical model (**c**) DCA of the combined model

This study constructed a radiomics model based on the selected optimal radiomic features. In the test set of five-fold cross-validation, the AUC values for the KNN, LR, and SVM algorithms were 0.860(95%CI:0.811–0.917), 0.916(95%CI:0.869–0.954), and 0.885(95%CI:0.817–0.931), respectively, as shown in the ROC curve in Fig. 4(a). In the clinical model based on independent clinical risk factors, the AUC values for the KNN, LR, and SVM algorithms were 0.868(95% CI: 0.798–0.912), 0.850(95% CI: 0.769–0.897), and 0.787(95% CI: 0.689–0.842), respectively, as shown in the ROC curve in Fig. 4(b). In the combined model based on clinical features and radiomic features, the AUC values for the KNN, LR, and SVM algorithms were 0.906(95% CI: 0.852–0.947), 0.954(95% CI: 0.919–0.982), and 0.930(95% CI: 0.891–0.964), respectively, as shown in the ROC curve in Fig. 4(c). The results of the DeLong test (see Supplementary Table S1) indicate that the differences in the AUC between the combined model and the single model for the LR and SVM algorithms are both statistically significant (*p* < 0.05). However, the difference in AUC between the KNN combined model and the radiomics model is not statistically significant (*p* > 0.05), while the difference in AUC between the KNN combined model and the clinical model is statistically significant (*p* < 0.05).The model calibration analysis (refer to Supplementary Figure S3 and Supplementary Table S2) demonstrated that the overall calibration accuracy of the combined model was significantly better than that of the single clinical model, as assessed by the Brier score. The results of the decision curve analysis (DCA) are presented in Fig. 5(a-c). The AUC differences among the models in the training set, validation set, and test set were all less than 0.1, aligning with the recommendations of the TRIPOD guidelines. The evaluation metrics for the training set are detailed in Table 3, the evaluation metrics for the validation set are presented in Table 4, and the evaluation metrics for each model in the test set are provided in Table 5.

**Table 3:** Performance indicators of each model in the training set

Model	Algorithm	AUC(95%CI)	Accuracy	Sensitivity	Specificity	F1 Score
Radiomics model	KNN	0.954(0.941–0.968)	0.866	0.922	0.804	0.878
	LR	0.928(0.906–0.949)	0.862	0.841	0.886	0.865
	SVM	0.893(0.851–0.910)	0.821	0.814	0.829	0.826
Clinical model	KNN	0.934(0.914–0.951)	0.866	0.857	0.875	0.870
	LR	0.843(0.807–0.869)	0.762	0.769	0.754	0.772
	SVM	0.788(0.735–0.811)	0.694	0.782	0.596	0.727
Combined model	KNN	0.957(0.944–0.969)	0.871	0.961	0.771	0.880
	LR	0.960(0.946–0.973)	0.867	0.857	0.879	0.871
	SVM	0.934(0.918–0.950)	0.840	0.870	0.807	0.851

*KNN* K-Nearest Neighbor; *LR* Logistic Regression; *SVM* Support Vector Machine; *AUC* Area Under the Curve

**Table 4:** Performance indicators of each model in the validation set

Model	Algorithm	AUC(95%CI)	Accuracy	Sensitivity	Specificity	F1 Score
Radiomics model	KNN	0.865(0.839–0.892)	0.767	0.838	0.689	0.791
	LR	0.910(0.886–0.932)	0.845	0.834	0.857	0.850
	SVM	0.851(0.819–0.879)	0.786	0.776	0.796	0.792
Clinical model	KNN	0.840(0.806–0.872)	0.760	0.718	0.807	0.758
	LR	0.827(0.792–0.858)	0.752	0.753	0.750	0.761
	SVM	0.755(0.715–0.795)	0.694	0.718	0.668	0.711
Combined model	KNN	0.882(0.856–0.907)	0.815	0.919	0.700	0.781
	LR	0.948(0.930–0.962)	0.861	0.841	0.882	0.862
	SVM	0.911(0.890–0.933)	0.830	0.847	0.811	0.839

*KNN* K-Nearest Neighbor; *LR* Logistic Regression; *SVM* Support Vector Machine; *AUC* Area Under the Curve

**Table 5:** Performance indicators of each model in the test set

Model	Algorithm	AUC(95%CI)	Accuracy	Sensitivity	Specificity	F1 Score
Radiomics model	KNN	0.860(0.811–0.917)	0.767	0.855	0.671	0.797
	LR	0.916(0.869–0.954)	0.843	0.830	0.857	0.846
	SVM	0.885(0.817–0.931)	0.809	0.804	0.814	0.814
Clinical model	KNN	0.868(0.798–0.912)	0.790	0.795	0.786	0.794
	LR	0.850(0.769–0.897)	0.762	0.794	0.729	0.773
	SVM	0.787(0.689–0.842)	0.694	0.704	0.686	0.702
Combined model	KNN	0.906(0.852–0.947)	0.829	0.921	0.729	0.760
	LR	0.954(0.919–0.982)	0.877	0.857	0.900	0.879
	SVM	0.930(0.891–0.964)	0.837	0.844	0.829	0.844

*KNN* K-Nearest Neighbor; *LR* Logistic Regression; *SVM* Support Vector Machine; *AUC* Area Under the Curve

## Discussion

This study for the first time established a combined model for diagnosing CF based on radiomics features of WMHs and independent risk factors. Compared to the clinical model based solely on independent risk factors and the radiomics model based on optimal radiomic features, the combined model demonstrates a higher clinical net benefit in diagnostic efficacy, a result validated by DCA. Among the KNN, LR, and SVM machine learning algorithms, the LR model exhibited the best overall performance, with superior calibration accuracy and clinical net benefit compared to the other algorithms. This may be attributed to the ability of LR to more effectively handle linear relationships between variables, resulting in better predictive performance. The advantage of the combined model may lie in the synergistic effect of radiomic features and clinical risk factors in reflecting white matter damage. Radiomic features can quantitatively characterize the microstructural heterogeneity of WMHs, such as lesion margin irregularity and internal texture complexity, which interact with clinical risk factors. Age is associated not only with increased WMH burden but also leads to more complex heterogeneity of WMHs by affecting the integrity of the blood-brain barrier and regulating cerebral blood flow [20]. In the case of diabetes, high blood glucose levels induce oxidative stress and vascular endothelial damage through metabolic abnormalities, while chronic inflammatory responses may disrupt the blood-brain barrier, exacerbating the progression of WMHs [21–23]. The interplay between these clinical risk factors and the microstructural characteristics of WMHs allows the combined model to more accurately assess the risk of cognitive decline from both macro- and micro-level perspectives.

In the radiomics model, the selected six optimal radiomic features include one first-order feature and five texture features. These features reflect the structural heterogeneity, homogeneity, and continuity of WMHs in the population by calculating statistical measures of inter-voxel correlations. Among these, the feature wavelet-HHH_firstorder_Energy (wHHHfE) has the highest feature coefficient and is the most important characteristic. Its significant positive regression coefficient indicates that this feature value is positively correlated with the risk of cognitive decline. As a first-order statistic calculated in the three-dimensional high-frequency subband (HHH) of wavelet transform, wHHHfE characterizes the intensity of high-frequency signals in local regions by computing the sum of squared voxel intensity values. An increase in this value, may reflect more significant microstructural damage in WMHs areas (such as edge sharpening or increased internal texture complexity). Notably, the sensitivity of this feature to lesion volume further underscores its clinical significance, as larger WMH volumes lead to higher energy values through cumulative effects, consistent with previous studies [10] that found “the overall volume of WMHs is associated with cognitive decline.” Therefore, wHHHfE not only serves as a powerful imaging biomarker for distinguishing cognitive decline but may also provide new quantitative indicators for assessing the extent of white matter damage, offering important imaging evidence for early identification and monitoring of cognitive decline.

In the clinical model, age, educational level, community activities, housework, clock-drawing test, and diabetes were identified as independent risk factors for CF, which is consistent with previous research findings [24–25]. As age increases, the body’s reserve capacity declines, and the hippocampus and cerebral cortex gradually atrophy, thus increasing the risk of CF. Additionally, studies have shown that receiving cultural education, participating in community activities, and engaging in housework can stimulate the nervous system, increase the number of neuronal dendrites and the thickness of the cerebral cortex, and inhibit the aggregation of β-amyloid in the brain, alleviating damage to cognitive function [26]. A higher score in the clock-drawing test indicates better integrity and accuracy in clock drawing [27]. Research has found that the clock-drawing test scores of frail elderly people are more than twice as low as those of non-frail elderly people [28]. Moreover, patients with diabetes are prone to chronic inflammation, which can interfere with insulin signaling and glutamate metabolism, damage glial cells and neurons, and subsequently lead to the occurrence of CF [29]. These clinical features provide important reference information for the diagnosis of CF.

In previous studies, Du J et al. [30] used the SPM12 software to calculate the volume of WMHs and analyzed the correlation between WMHs volume and cognitive frailty by measuring the WMHs volume. Zheng W et al. calculated the WMHs volume using the LR model and explored metabolic-syndrome-related cognitive impairment based on graph-theory analysis [31]. However, these methods failed to fully uncover the potential information within WMHs. The advantage of this study lies in the use of radiomics to analyze the imaging information in WMHs images that is difficult to be recognized by the human eye, and the establishment of a combined model by integrating clinical features. Compared with studies that constructed a diagnostic model for CF using only clinical features [32], the combined model significantly improved the diagnostic performance (AUC value of the combined model was 0.954, while that of the model constructed using only clinical features was 0.889).

This study has certain limitations: (1) The semi-automated delineation method for WMHs used in the research resulted in a longer processing time. Future studies will consider incorporating deep learning methods or using segmentation software to achieve automatic segmentation of WMHs, thereby avoiding subjective errors introduced by manual delineation and improving analysis efficiency and accuracy. (2) This study is a single-center research conducted in the Chuan-Yu region, where the population exhibits significant regional characteristics in dietary habits. Such regional differences in dietary structure may affect the predictive performance of diabetes-related metabolic markers, thus limiting the generalizability of the model to other populations. Future efforts will focus on enhancing the model’s generalization ability by increasing the sample size and conducting multicenter studies. (3) The data were derived from a cross-sectional study conducted over a specific time period, which does not allow for the analysis of the relationship between radiomic features and disease progression. We will continue to collect patient outcome events to capture individual changes over time. (4) The size of the test set in this study is limited. In the future, external validation will be carried out by collecting data from other hospitals.

## Conclusions

This study demonstrates that models constructed using KNN, LR, and SVM algorithms, based on quantitative analysis of WMHs through radiomic techniques, exhibit good diagnostic efficacy for CF. Further analysis revealed that combining clinical independent risk factors with radiomic features can enhance the clinical decision-making value and calibration accuracy of the model. This diagnostic model provides an objective and precise tool for the early identification of CF, assisting clinicians in formulating timely personalized intervention strategies to delay disease progression and improve patient prognosis and quality of life.

## Electronic supplementary material

Below is the link to the electronic supplementary material.

Supplementary Material 1

## Data Availability

The data was available from the corresponding author with reasonable request.

## Abbreviations

CF: Cognitive frailty
WMHs: White matter hyperintensities
MRI: Magnetic resonance imaging
T2–FLAIR T2: Fluid-attenuated inversion-recovery
MMSE: Mini-Mental State Examination
FP: Fried frailty phenotype
ROI: Region of interest
ICC: Intraclass Correlation Coefficient
LASSO: Least Absolute Shrinkage and Selection Operator
LR: Logistic Regression
AUC: Area under the curve
KNN: K-nearest neighbor
SVM: Support vector machine
DCA: Decision curve analysis
CAD: Coronary Artery Disease
CDT: Clock Drawing Test
OR: Odds ratio
CI: Confidence interval
AUNBC: Area under the net benefit curve
