# Supplementary material for: The value of radiomics features of white matter hyperintensities in diagnosing cognitive frailty: a study based on T2-FLAIR imaging
Source: BMC Med Imaging. 2025 May 22;25:181. doi: 10.1186/s12880-025-01732-y (PMC12100808; doi:10.1186/s12880-025-01732-y)

**Supplementary** **file 3 Sample size**：In this study, the pwr package in R software (version 4.3.2) was used to calculate the sample size. Cohen's d was set at 0.5 (moderate effect), the significance level (α) was set at 0.05, and the statistical power (1-β) was set at 0.8. Through calculation, at least 64 subjects should be included in each group, and the total sample size was 128 cases. In this study, a total of 147 patients were included (77 cases with CF and 70 cases in the control group), which met the requirement of the minimum sample size. Furthermore, in this study, six radiomic features were incorporated. Each predictor variable corresponded to 12.8 events (with 77 events, EPP = 77/6 = 12.8), which met the empirical criterion that at least 10 events per parameter (EPP ≥ 10) are required for the development of the predictive model. This indicates that the ratio of the sample size to the radiomics features is appropriate, ensuring the reliability of the model development.

**Supplementary Figure S1.** Schematic diagram of participant selection process for the study.


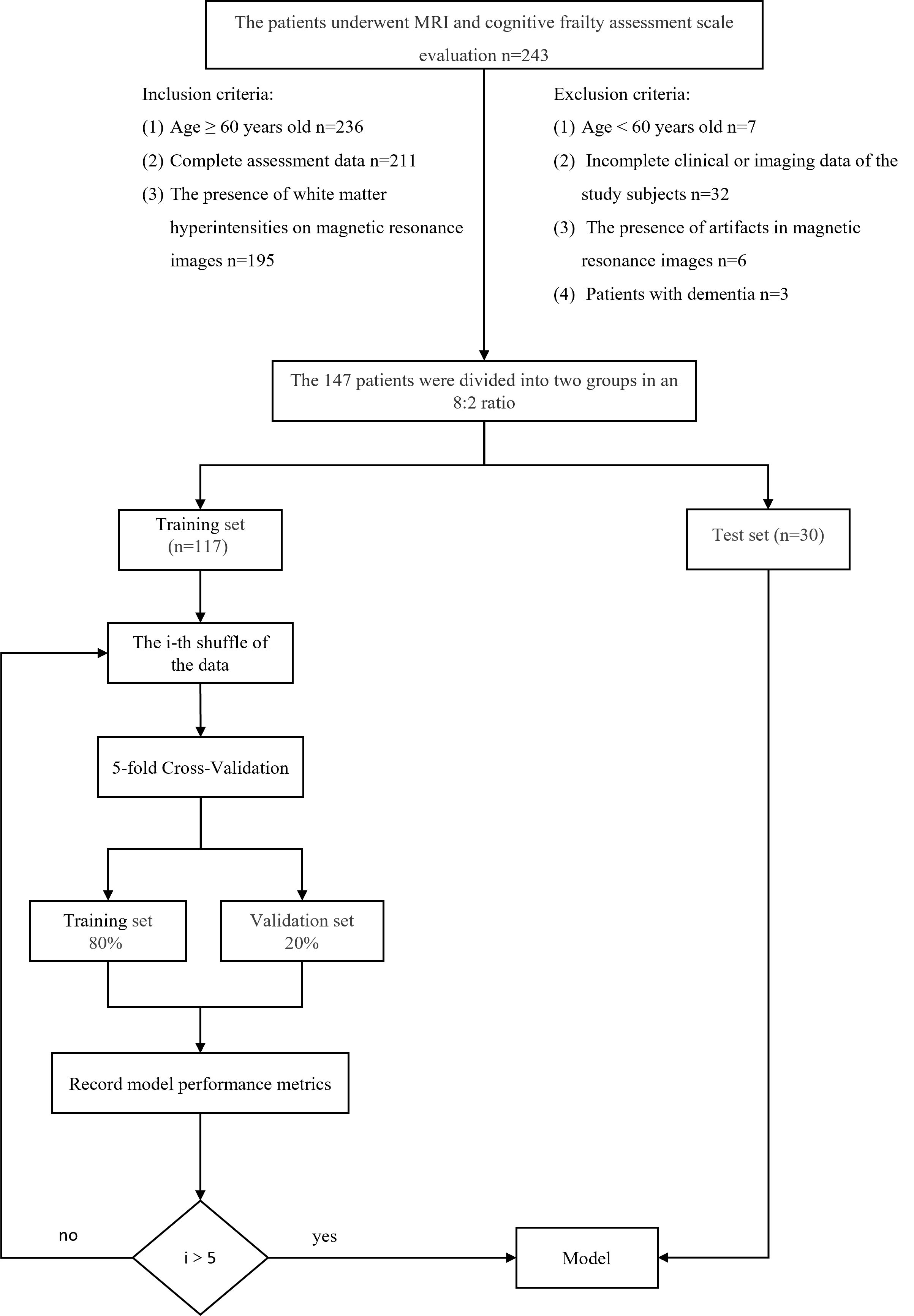


**Supplementary Figure S2.** **ROI delineation** **(a-c) Cognitive frailty case (d-f) Non-cognitive frailty case**


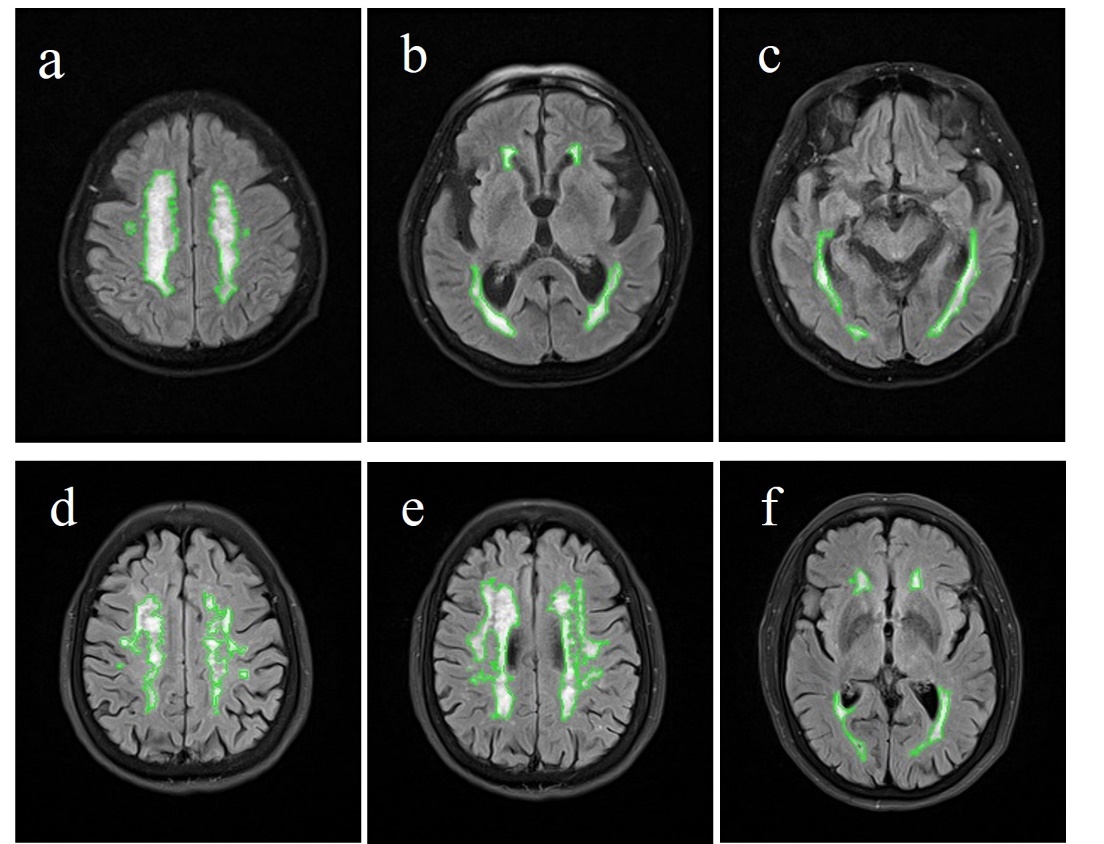


**Supplementary Table S1** Results of DeLong Test for Each Model

| Model | Z | *p* |
| --- | --- | --- |
| Radiomics model |  |  |
| KNN VS LR  KNN VS SVM  LR VS SVM  Clinical model | -2.187  -0.424  2.713 | 0.029*  0.672  0.007* |
| KNN VS LR  KNN VS SVM  LR VS SVM  Combined model | 0.852  3.040  5.830 | 0.394  0.002*  ＜0.001* |
| KNN VS LR  KNN VS SVM  LR VS SVM | -2.699  -1.685  1.837 | 0.007*  0.092  0.662 |
| Radiomics model VS Clinical model |  |  |
| KNN | 0.225 | 0.822 |
| LR | 1.974 | 0.048* |
| SVM | 0.378 | 0.017* |
| Combined model VS Radiomics model |  |  |
| KNN | 1.205 | 0.228 |
| LR | 2.123 | 0.034* |
| SVM | 2.237 | 0.025* |
| Combined model VS Clinical model |  |  |
| KNN | 2.325 | 0.020* |
| LR | 2.412 | 0.016* |
| SVM | 5.846 | ＜0.001* |

*KNN* K-Nearest Neighbor *LR* Logistic Regression *SVM* Support Vector Machine ******p*＜0.05

**Supplementary Table S2** Brier Scores of Different Models

| Model | Algorithm | Training set | Validation set | Test set |
| --- | --- | --- | --- | --- |
| radiomics model | KNN | 0.085 | 0.147 | 0.148 |
|  | LR | 0.108 | 0.120 | 0.118 |
|  | SVM | 0.155 | 0.162 | 0.144 |
| clinical model | KNN | 0.107 | 0.165 | 0.156 |
|  | LR | 0.167 | 0.174 | 0.169 |
|  | SVM | 0.196 | 0.201 | 0.194 |
| combined model | KNN | 0.099 | 0.141 | 0.135 |
|  | LR | 0.096 | 0.104 | 0.097 |
|  | SVM | 0.105 | 0.120 | 0.106 |

*KNN* K-Nearest Neighbor *LR* Logistic Regression *SVM* Support Vector Machine

**Supplementary Figure S3.** Calibration curves of different models in the training, validation, and test sets (a-c), Calibration curves of radiomics models (d-f), Calibration curves of clinical models (g-i), Calibration curves of combined models.


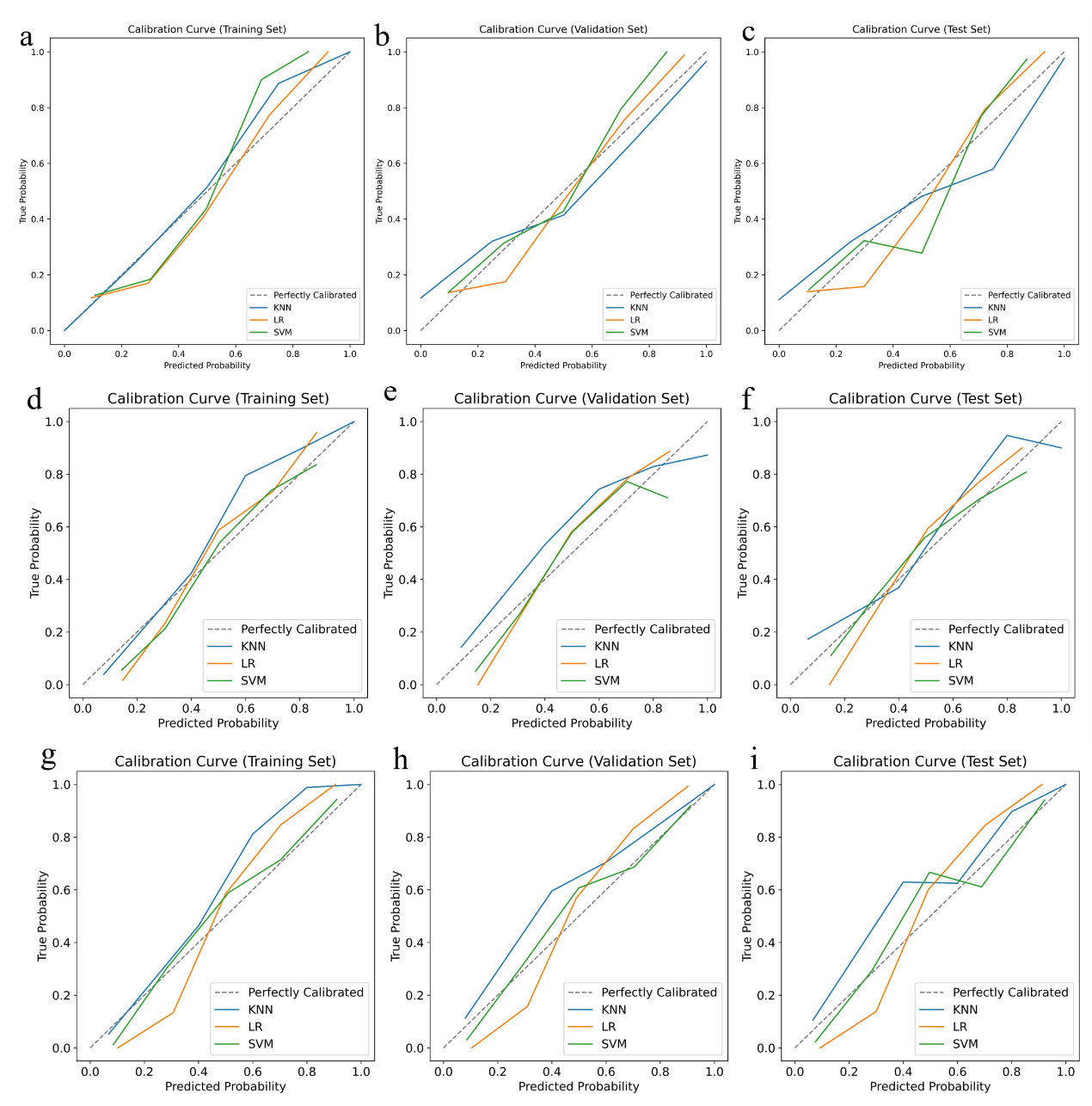


**Supplementary file 4** PyRadiomics configuration file


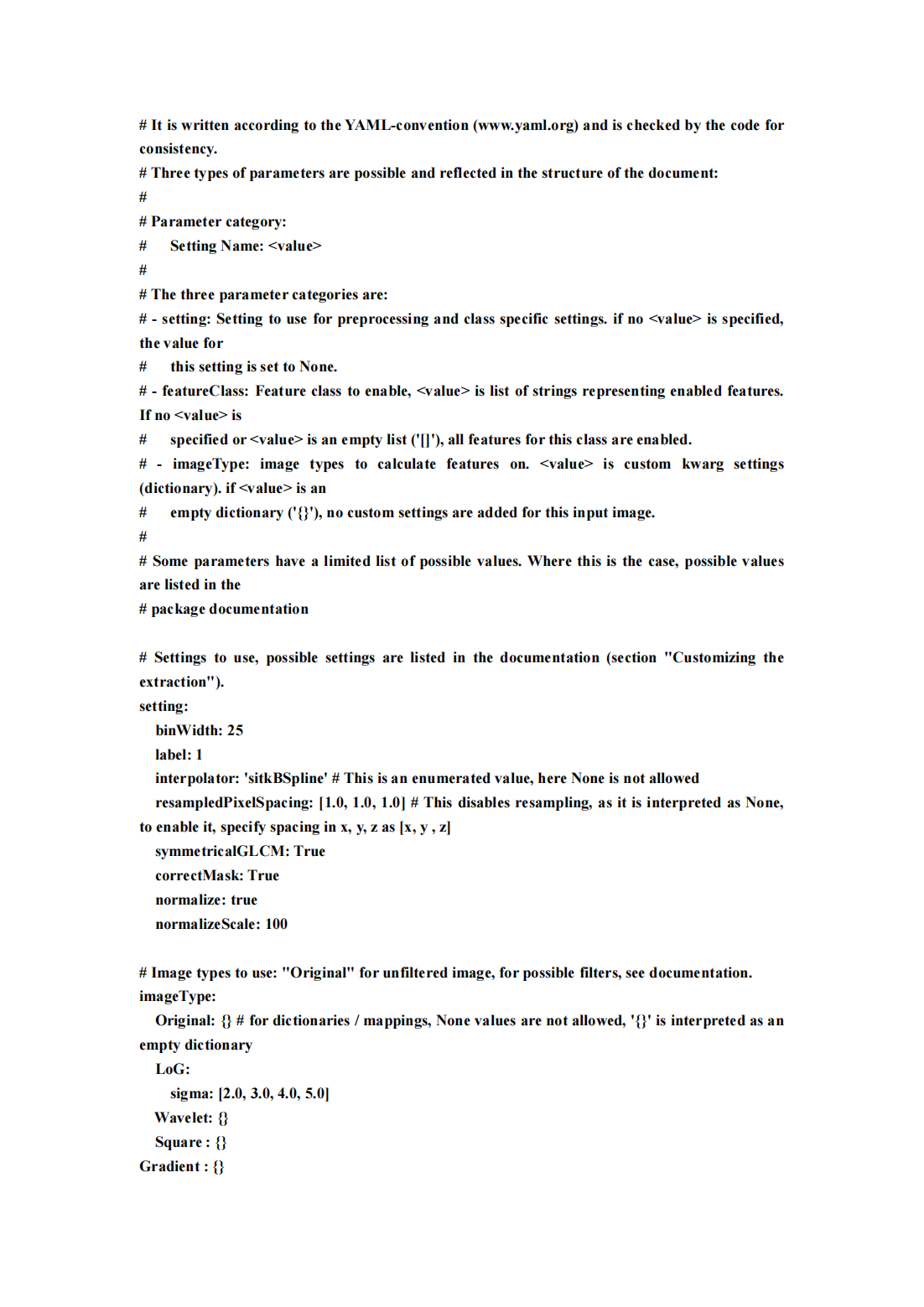


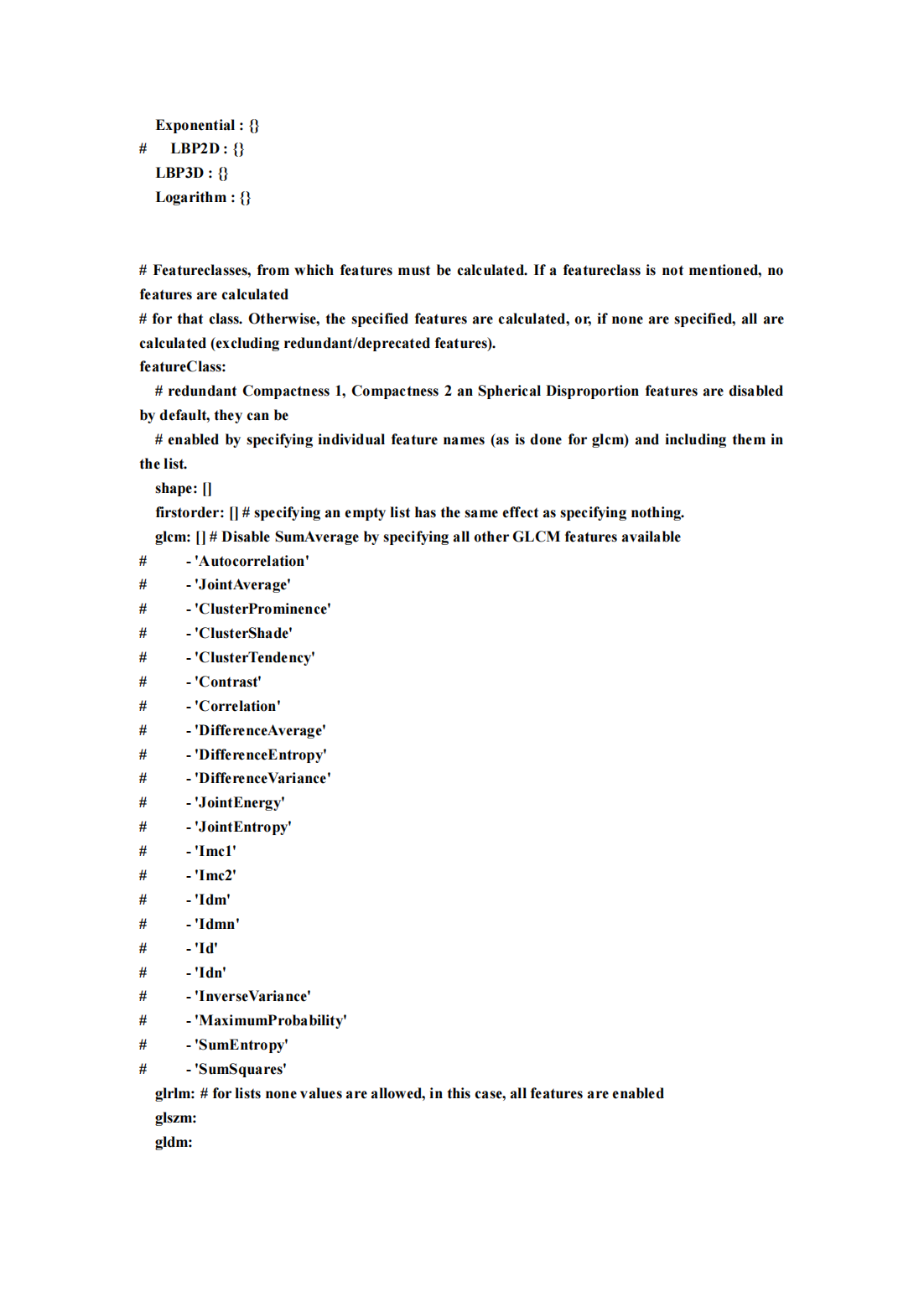

Supplement: Supplementary file 1 — Supplementary Material 1 [file 12880_2025_1732_MOESM1_ESM.docx]
